# A New Vanadium (III) Complex of 2,6-Bis(3,5-diphenylpyrazol-1-ylmethyl)pyridine as a Catalyst for Ethylene Polymerization

**DOI:** 10.3390/molecules18044728

**Published:** 2013-04-22

**Authors:** Hanna S. Abbo, Salam J. J. Titinchi

**Affiliations:** Department of Chemistry, University of the Western Cape, Private Bag X17, Bellville 7535, Cape Town, South Africa; E-Mail: stitinchi@uwc.ac.za

**Keywords:** pyrazole, 2,6-bis(pyrazol-1-ylmethyl)pyridine, vanadium complex, ethylene polymerization, high density polyethylene

## Abstract

The tridentate ligand 2,6-bis(3,5-diphenylpyrazol-1-ylmethyl)pyridine, abbreviated as 2,6-[(3,5-ph_2_pz-CH_2_)_2_-py], a new pyridine-pyrazole derivative, was prepared from 2,6-bis(bromomethyl)pyridine and 3,5-diphenylpyrazole. The ligand was characterized by means of elemental analyses, ATR-IR, ^1^H- and ^13^C-NMR spectroscopy and single crystal X-ray crystallography. Using this ligand, a new mononuclear vanadium (III) complex, {2,6-[(3,5-ph_2_pz)CH_2_]_2_py}VCl_3_ was prepared and characterized by elemental analysis, ATR-IR and HR-MS. The complex was investigated as a catalyst for ethylene polymerization and compared with 2,6-[(3,5-Me_2_pz)CH_2_]_2_py}VCl_3_ to explore the effect of the substituent on the pyrazolyl rings on ethylene polymerization. High catalytic activity was observed at ambient temperature. After treatment with AlEtCl_2_, these complexes showed high activity for ethylene polymerization converting ethylene to highly linear polyethylene and affording high molecular weight polymers (up to 1.0 × 10^6^ g/mol) with unimodal molecular weight distributions.

## 1. Introduction

Pyrazole-based ligands are widely used in syntheses of transition metals coordination compounds, which has been reviewed [1]. They can act as terminal or bridging ligands and have tridentate chelation at each metal centre [2,3]. Despite the numerous reports on the synthesis and properties of late transition metal complexes containing pyrazolyl based ligands, very little is known about their catalytic activity in olefin oligomerization and polymerization [4,5,6,7,8,9]. 

An interesting fact arises when a six-membered heterocycle such as pyridine and a five-membered heterocycle such as pyrazole are combined in a single ligand unit. The substitution at the 2 and 6-positions of the pyridine ring gives tridentate ligands such as 2,6-bis(pyrazol-1-yl)pyridine and 2,6-bis(pyrazol-1-ylmethyl)pyridine. The coordination chemistry of pyrazolyl-pyridine-based chelating ligands has been studied extensively [10,11,12,13,14]. The possibility of introducing groups in the 3,5-positions of the pyrazole ring causes tuning of the electronic and steric properties, depending on the substituents used, which modify the catalytic response of the metal center and define the polymerization reaction. The structure of these tridentate ligands is closely related to the 2,6-diiminopyridine [15], which have been ubiquitously used as ligands for post-metallocene olefin polymerization catalysts.

In recent years, there has been considerable interest in designing new catalysts of late transition metal complexes consisting of tridentate nitrogen-based ligands as non-metallocene olefin polymerization catalysts [15,16]. These catalysts enhance the both activity and microstructure of the resultant polymers.

To expand our research work [6,9], which aims to synthesize new pyrazolyl-pyridine complexes with high catalytic activity towards ethylene polymerization, herein we describe the synthesis of the ligand 2,6-[(3,5-ph_2_pz-CH_2_)_2_-py] and its mononuclear vanadium (III) complex, {2,6-[(3,5-ph_2_pz)CH_2_]_2_py}VCl_3_. We have also studied the influence of polymerization conditions, such as temperature, pressure, reaction time and optimized Al/V molar ratios on the productivity of polyethylenes and on the polyethylene properties. To our knowledge the vanadium complex reported herein is amongst the first examples of a vanadium pyrazolyl-pyridine catalyst bearing phenyl groups as a precursor for ethylene polymerization which shows long lifetime during the catalytic cycle.

## 2. Results and Discussion

### 2.1. Synthesis of 2,6-Bis(3,5-diphenylpyrazol-1-ylmethyl)pyridine and Its Vanadium Complex

**Scheme 1:**
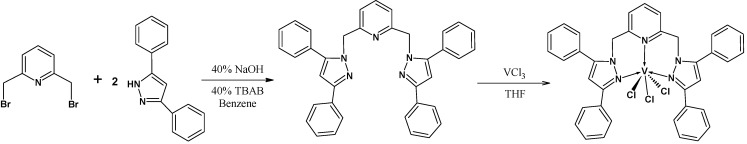
Synthesis of 2,6-[(3,5-ph_2_pz-CH_2_)_2_-py] and its vanadium complex **1**.

**Figure 1:**
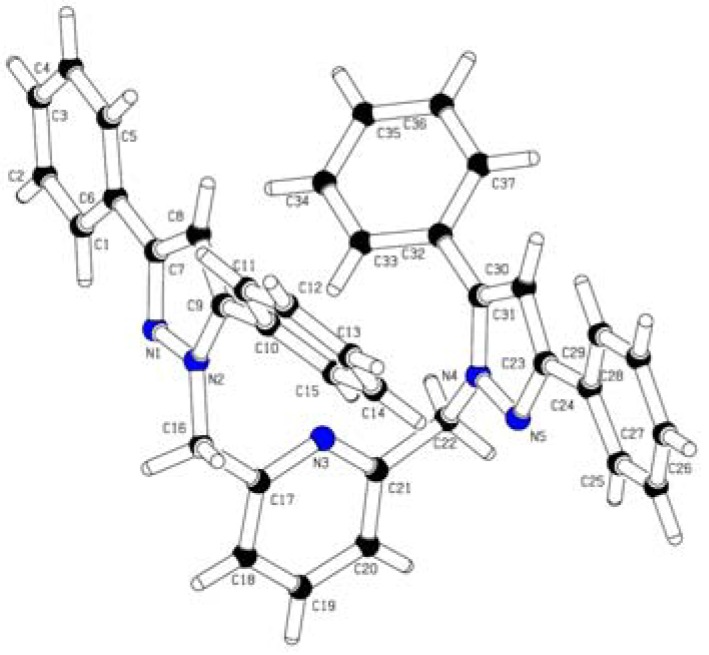
Molecular structure of 2,6-[(3,5-ph_2_pz-CH_2_)_2_-py].

2,6-Bis(3,5-diphenylpyrazol-1-ylmethyl)pyridine was synthesized by the reaction of two equivalents of 3,5-diphenylpyrazole and one equivalent of 2,6-bis(bromomethyl)pyridine in benzene in the presence of 40% aqueous NaOH and 40% aqueous tetrabutylammonium bromide (Scheme 1). Analytically pure compounds were obtained in 78% yield after purification by chromatography on silica gel. This compound is very stable in air and can be stored at room temperature for a long period of time. It was characterized by NMR and IR spectroscopy, as well as by microanalysis and X-ray crystallography. The proton and ^13^C-NMR and the IR data, along with elemental analysis for the ligand correspond very well with its crystal structure. Crystallographic data for the structure reported here has been deposited with CCDC (Deposition No. CCDC-688192; CCDC 688192 contains the supplementary crystallographic data for this paper. These data can be obtained free of charge via www.ccdc.cam.ac.uk/conts/retrieving.html, or from the Cambridge Crystallographic Data Centre, 12 Union Road, Cambridge CB2 1EZ, UK; fax: +44 1223 336 033; or e-mail: deposit@ccdc.cam.ac.uk). The crystal data and structural refinement parameters of the title compound are summarized in Table 1. The molecular structure and the numbering for the ligand atoms is shown in Figure 1. 

**Table 1:** Crystal data and structural refinement parameters of 2,6-[(3,5-ph_2_pz-CH_2_)_2_-py].

**CCDC deposition**	**688192**	
Empirical formula	C_37_H_29_N_5_	
Formula weight	543.65	
Temperature	298(2) K	
Wavelength	0.71073 Å	
Crystal system	Orthorhombic	
Space group	Pbca	
Unit cell dimensions	a = 16.5531(4) Å	α = 90°.
	b = 9.5085(2) Å	β = 90°.
	c = 37.3606(8) Å	γ = 90°.
Volume	5880.4(2) Å^3^	
Z	8	
Density (calculated)	1.228 Mg/m^3^	
Absorption coefficient	0.074 mm^−1^	
F(000)	2288	
Crystal size	0.32 × 0.14 × 0.12 mm^3^	
Theta range for data collection	1.09 to 28.00°.	
Index ranges	−20<=h<=21, −12<=k<=11, −48<=l<=49	
Reflections collected	68614	
Independent reflections	7105 [R(int) = 0.1090]	
Completeness to theta = 28.00°	100.0%	
Absorption correction	None	
Max. and min. transmission	0.9912 and 0.9768	
Refinement method	Full-matrix least-squares on F^2^	
Data/restraints/parameters	7105/0/380	
Goodness-of-fit on F^2^	0.670	
Final R indices [I>2sigma(I)]	R_1_ = 0.0400, wR_2_ = 0.0928	
R indices (all data)	R_1_ = 0.1564, wR_2_ = 0.1467	
Extinction coefficient	0.0021(3)	
Largest diff. peak and hole	0.229 and −0.180 e^−3^ Å	

The results of the ^1^H-NMR, IR and elemental analysis were in good agreement with the proposed structure of the title compound. The ORTEP structure shows that the two pyrazolyl units are not coplanar with the pyridine ring and form dihedral angles N(2)-C(16)-C(17)-N(3) of −33.3(2)° and N(4)-C(22)-C(21)-N(3) of −41.6(2)°. It can be found that the carbon–nitrogen bond lengths on the molecular skeleton are basically intermediate between typical C-N single (1.47 Å) and C=N double bonds (1.27 Å). The two substituted pyrazolyl units and their phenyl groups are pointed outwards from the pyridine ring. The phenyl rings attached to carbon C7 and C23 are not parallel but not quite coplanar with the pyrazolyl ring, with dihedral angles C(1)-C(6)-C(7)-N(1) of 6.5(3)° and C(25)-C(24)-C(23)-N(5) of −15.3(3)°, respectively. On the other hand, the phenyl rings attached to C9 [C(10)-C(15)] and C31 [C(32)-C(37)] are tilted by 131.3(2)° and −46.7(3)°, with dihedral angles of N(2)-C(9)-C(10)-C(11) and N(4)-C(31)-C(32)-C(33), respectively, with respect to the pyrazolyl rings.

The vanadium complex, {2,6-[(3,5-ph_2_pz)CH_2_]_2_py}VCl_3_, was prepared in good yield (65%) by the reaction of this tridentate ligand with VCl_3_ (Scheme 1). The complex was characterized by a combination of ATR-IR spectroscopy, high resolution mass spectrometry and micro analysis. 

A strong band at 1574 cm^−1^ in the spectrum of the ligand assigned to ν(C=N) was lowered by 20 cm^−1^ upon complexation due to the coordination of the azomethine nitrogen with the metal ion. The complex exhibited band at 378 cm^−1^ due to ν(V-Cl) stretching mode [17]. 

The mass spectrum of the complex showed the molecular ion and the base peak at m/z 698.15 [M−H] and 544.25 [M-VCl_3_], respectively, confirming its formula. The mass fragmentation pattern supported the suggested structure of the complex. The mass and microanalytical data of the complexes correspond well with the proposed structure. The coordination between the N-pyridine moiety and the metal ion were confirmed by crystal structures for related systems [8,15,18]. No suitable NMR data were obtained due to the paramagnetism of the metal used. Repeated attempts to grow an X-ray crystal of the complex were unsuccessful.

### 2.2. Polymerization of Ethylene

On the basis of the encouraging preliminary reported results using 2,6-[(3,5-Me_2_pz-CH_2_)_2_-py]VCl_3_, (**2**), as an active catalyst for ethylene polymerization [6], it was selected for further optimization, investigating the influence of polymerization conditions on the productivity of the catalyst system employed and the nature of the polymer produced. These results promote us to prepare 2,6-[(3,5-ph_2_pz-CH_2_)_2_-py]VCl_3_ (**1**) as a new catalyst for ethylene polymerization and to explore the effect of the substituent on the pyrazolyl rings on the ethylene polymerization outcome.

Ethylene polymerization studies were conducted in toluene using a 300 mL stainless steel Parr reactor. To study the influence of the catalytic polymerization conditions on the productivity of the catalysts employed, we carried out a series of experiments under different reaction times, temperature, pressure, as well as the ratio of Al/V. Table 2 shows the optimization results for the ethylene polymerization using catalysts **1** and **2** and its influence on the polyethylene properties produced.

**Table 2:** Polymerization of ethylene using precatalysts **1** and **2**.

**Run No.**	**Cat(μmol)**	**Al/V ratio**	**Temp(°C)**	**Time(min)**	***P* C_2_H_4_(bar)**	**YieldPE/g**	**Activity(Kg mol^−1^·h^−^^1^)**	**M_w_×10^−6^**	**PD**
1	1(2.5)	1500	25	30	10	1.25	1000	0.89	2.7
2	1(2.5)	1500	50	30	10	0.38	304	0.73	4.3
3	1(1.5)	1500	25	15	10	0.59	1570	1.0	3.1
4	1(1.5)	1500	25	30	10	0.99	1320	0.92	2.8
5	1(5)	1500	25	60	10	1.27	270	--	--
6	1(1.5)	1000	25	30	10	0.47	626	--	--
7	1(1.5)	2000	25	15	10	0.21	560	--	--
8	1(2.5)	1500	25	15	1	0.17	272	0.74	4.3
9	2(5)	500	25	10	10	2.0	2400	0.6	2.9
10	2(5)	500	25	60	10	4.3	860	---	---
11	2(5)	250	25	10	10	2.5	3010	0.8	2.2
12	2(5)	250	25	30	10	3.1	1240	--	---
13	2(5)	250	50	30	10	0.73	292	--	--
14	2(5)	250	25	60	10	4.7	940	0.68	2.5
15	2(2.5)	500	25	30	10	1.7	1360	--	---
16	2(2.5)	500	25	60	10	3.5	1400	0.58	2.4
17	2(5)	1000	25	60	10	4.2	840	0.59	2.3
18	2(5)	1000	25	120	10	5.6	560	--	--
19	2(5)	250	25	10	1	0.35	422	0.75	3.2
20	2(5)	250	25	60	1	2.1	410	0.6	2.5

Upon activation with a small amount of AlEtCl_2_, both complexes show good catalytic activity for ethylene polymerization under 1 and 10 bar of ethylene. The polyethylenes produced with these systems have a high molecular weight (Mw) between 0.6 − 1.0 × 10^6^ g/mol and relatively narrow molecular weight distributions (M_w_/M_n_ = 2.2 − 4.3) in all cases, depending on reaction conditions. This might suggest that the polymerization proceeds via a single-site.

Both catalysts are most active at 25 °C, with maximum productivity of 1,570 Kg·mol^−1^·h^−1^ for catalyst **1** and 3,010 Kg·mol^−1^·h^−1^ for catalyst **2**. At 50 °C the overall productivity drops substantially for both catalysts after 30 min reaction time (entries 2 and 13) due to partial decomposition of the catalytically active species at higher temperatures. This observation promises advantages for industrial use of these catalysts, as commercial ethylene polymerization is mainly done at high temperature and pressure. The polymer molecular weight tends to decrease with the increasing temperature of polymerization, which could be attributed to the much faster β-hydride elimination over the propagation.

To investigate the effect of co-catalyst to catalyst ratio, the Al/V ratio was varied from 1,000:1 to 2,000:1 for catalyst **1** and the optimum ratio was found to be 1,500:1 (entry 3), while for catalyst **2**, theoptimum ratio of Al/V ratio is 250:1 (entry 11). The possible reason is that an optimal amount of AlEtCl_2_ is needed to effectively activate the catalyst, while an excess amount will create too many alkylaluminum impurities and may cause the degradation of the catalyst system.

High catalyst concentration results in a decrease of the catalytic activity for both catalysts, which could be interpreted as a mass-transport effect, moreover the reaction mixture becomes denser at higher concentration, which inhibits ethylene from reaching the active center. 

The observed activity decreased at lower ethylene pressure 1 bar and increasing the pressure to (10 bar) increased the activity under same conditions for both catalysts (entries 8, 19 and 20), probably as an effect of increased ethylene concentration in solution.

The reaction time has a great effect on productivity for vanadium-based polymerization catalysts as they are generally considered to deactivate through reduction of the metal centre to V (II) during the catalytic cycle [19,20,21,22,23]. A series of experiments were carried out at 25 °C for range of time 10–60 min, thus, increasing the reaction time from 15 min to 1 h, the activity for **1** was observed to decrease dramatically (entries 3 and 5). The activity for **2** on the other hand, decreased from 3,010 to 940 Kg·mol^−1^·h^−1^ when the reaction time increased from 10 min to 1 h (entries 11 and 14). This may be attributed to the embedding of catalyst sites in the solid polyethylene produced during the first minutes. In comparison to the earlier reports for known vanadium systems which usually deactivated after short time under polymerization conditions, our catalysts are more stable and still have moderate catalytic activity after 30 min. [19,20,21,22,23]. It appears that the ligand system that we are employing is capable of stabilizing the activated metal centre. 

The polymerization activity of a catalyst can be influenced by the steric and electronic characteristics of the ligand. Catalyst **2** bearing electron-donating methyl groups on the pyrazolyl ring exhibited higher activity than the corresponding catalyst **1** bearing electron-withdrawing phenyl groups. A possible reason for decreasing in activity with increased steric bulk could be reduced accessibility of the monomer to the vacant metal center. On the other hand, it is commonly believed that electron-donating groups increased the stability of the active species while electron-withdrawing groups reduce the stability of the active species.

**Figure 2:**
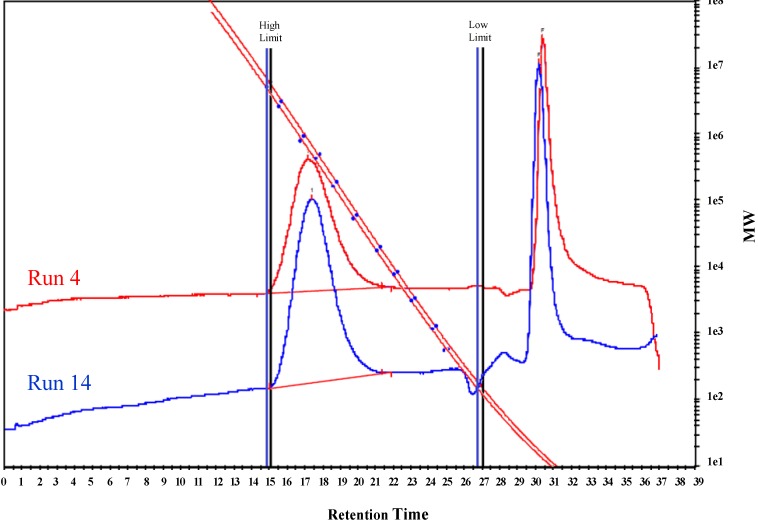
GPC diagram of polyethylene provided by runs 4 and 14.

**Figure 3:**
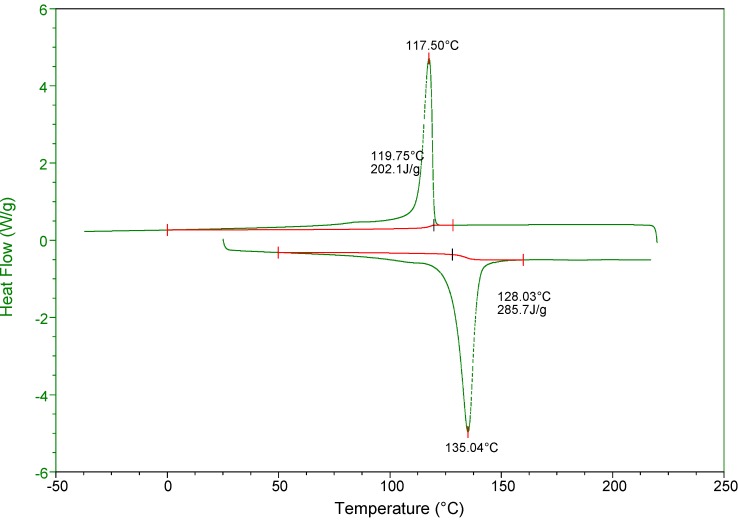
DSC profile of polyethylene sample produced by catalyst **1** (entry 1).

Gel permeation chromatography (GPC) analyses (Figure 2) of the polyethylenes obtained indicate that they possess high molecular weights with narrow molecular weight distributions in all cases as expected for single-site catalysts. The high–temperature NMR spectra verified that all polyethylenes produced by both catalysts are of high order linearity, which based upon the observation of only signal from the methylene group (−CH_2_−) in ^13^C-NMR spectra (Figure 1S). Differential scanning calorimetry (DSC) analyses demonstrated that the polyethylenes are essentially linear and show melting points of ca. 135 °C (Figure 3).

## 3. Experimental

### 3.1. General

All reagents were of reagent grade and were used without further purification. The NMR spectra were recorded on a Varian Gemini 2000 instrument (^1^H at 200 MHz and ^13^C at 50.1 MHz) at room temperature for the ligand in CDCl_3_, while those of polyethylene were recorded in 1,2,4-trichlorobenzene/benzene-d_6_ at 120 °C. Elemental analysis was performed by the micro analytical laboratory at the University of Cape Town, South Africa. FT-IR spectra were carried out on a Perkin-Elmer Spectrum 100 FTIR spectrometer. High resolution mass spectra were determined on a Waters Synapt G2 mass spectrometer (ASAP probe and ESI) at the Central Analytical Facility, University of Stellenbosch (South Africa). Polymer molecular weights were performed by high temperature GPC (1,2,4-trichlorobenzene, 160 °C, rate = 1.0 mL/min) on a Polymer Laboratories GPC220 instrument using polystyrene standards at the Institute of Polymer Science at the University of Stellenbosch (South Africa). Crystallographic data was collected on a Bruker APEX II CCD area detector diffractometer with graphite monochromated Mo *K*_α_ radiation (50kV, 30mA) using the APEX 2 [24] data collection software. The collection method involved ω-scans of width 0.5° and 512 × 512 bit data frames. Data reduction was carried out using the program *SAINT+* and absorption corrections were made using *XPREP* [25]. The crystal structure was solved by direct methods using *SHELXTL* [26]. Non-hydrogen atoms were first refined isotropically followed by anisotropic refinement by full matrix least-squares calculations based on *F*^2^ using *SHELXTL*. Hydrogen atoms were first located in the difference map then positioned geometrically and allowed to ride on their respective parent atoms. Diagrams and publication material were generated using SHELXTL, PLATON [27] and ORTEP-3 [28].

### 3.2. Synthesis of 3,5-Diphenylpyrazole

This compound was synthesized following a procedure described in the literature [29] with slight modifications. To a well stirred solution of dibenzoylmethane (5 g, 22.32 mmol) in (50 mL) ethanol, hydrazine monohydrate (1.74 mL, 35.57 mmol) was added dropwise. The reaction mixture was heated at 50 °C for ½ h, and then refluxed overnight and white crystals were obtained, filtered, washed with a small volume of ethanol and dried in air.

### 3.3. Synthesis of 2,6-[(3,5-ph2pz-CH2)2-py]

To a well stirred solution mixture of 2,6-bis(bromomethyl)pyridine (0.5 g, 1.89 mmol) and 3,5-diphenylpyrazole (0.83 g, 3.77 mmol) in benzene (40 mL) was added dropwise 40% aqueous NaOH (12 mL) and 40% aqueous tetrabutylammonium bromide (12 drops) (Scheme 1). The reaction mixture was heated under reflux for 18 h. The organic layer was separated, dried over MgSO_4_, filtered and concentrated by distillation under reduced pressure. Pure product (0.8 g, 78%) was obtained by column chromatography over silica gel (CH_2_Cl_2_/ether, 8:1) as a white solid. Anal. Calc. for C_37_H_29_N_5_ (543): C, 81.76; H, 5.34; N, 12.89. Found: C, 81.06; H, 5.25; N, 12.23%. ^1^H-NMR (CDCl_3_): 5.47 (4H, s, CH_2_); 6.69 (2H, s, 4-pz); 6.88 (2H, d, 3,5-py); 7.34–7.45 (16H, m, 2,3,5,6-ph); 7.58 (1H, t, 4-py); 7.86 (4H, dd, 4-ph); ^13^C-NMR (CDCl_3_): δ 54.33; 103.75; 120.46; 125.82; 127.98; 128.64; 128.78; 130.01; 146.32; 151.35; 156.76. IR: (KBr cm^−1^): *ν*(C=N) 1574 cm^−1^. High-quality off-white single crystals suitable for X-ray diffraction were obtained by recrystallization from ethanol.

### 3.4. Synthesis of {2,6-[(3,5-ph2pz)CH2]2py}VCl3 (**1**)

A suspension of VCl_3_ (0.062 g, 0.34 mmol) in anhydrous THF (10 mL) was added dropwise to a stirred solution of 2,6-[(3,5-ph_2_pz-CH_2_)_2_-py] (0.213 g, 0.34 mmol) (Scheme 1). Upon stirring and heating to 65 °C the colour of the turbid reaction mixture changed to greenish gray after 2 h. After 24 h and having cooling down the reaction medium to room temperature, it was concentrated to ca. 2 mL by rotary evaporation. Addition of hexane at this stage precipitated a dark greenish gray powder from the solution. It was filtered, washed with hexane and ether, and dried under vacuum. Yield: 0.11 g (46%), M.p. > 300 °C. Anal. (C_37_H_29_N_5_Cl_3_V) Calcd: C, 63.40; H, 4.14; N, 9.99. Found: C, 62.50; H, 4.55; N, 9.93%. IR (KBr cm^−1^): (C=N) 1554 cm^−1^. HRMS-TOF (ESI+) (ASAP): *m/z* 698.15 [M-H]; 628.16 [M-Cl_2_]; 544.25 [M-VCl_3_] (base peak); 219.09 [3,5-diphenylprazolyl, C15H11N2] (Figure 2S).

## 4. Conclusions

The vanadium complex reported herein is amongst the first example of a vanadium pyrazolyl-pyridine catalyst bearing phenyl groups useful as a catalyst precursor for ethylene polymerization. Moreover, further studies on this catalytic system have been explored, including the effect of reaction parameters and the effect of substituents on the pyrazolyl ring on the catalyst activity performance.

In the presence of AlEtCl_2_, pyrazolyl-pyridine vanadium complexes were highly active catalysts for ethylene polymerization at 25 °C, producing high molecular weight polyethylenes with narrow molecular weight distributions under all types of polymerization conditions. These results indicate that the pyrazolyl-pyridine ligand system is capable of stabilizing the active species and maintaining single-site catalytic behaviors of the vanadium catalysts for ethylene polymerization. In addition, these catalysts show extended catalyst life times compared with known vanadium systems which are usually deactivated after a short time under the typical polymerization conditions.

## Supplementary Materials

Supplementary materials can be accessed at: http://www.mdpi.com/1420-3049/18/4/4728/s1.
