# Supplementary material for: A New Vanadium (III) Complex of 2,6-Bis(3,5-diphenylpyrazol-1-ylmethyl)pyridine as a Catalyst for Ethylene Polymerization
Source: Molecules. 2013 Apr 22;18(4):4728–38. doi: 10.3390/molecules18044728 (PMC6270057; doi:10.3390/molecules18044728)
Supplement: Supplementary file 1 [file molecules-18-04728-s001.pdf]

Supplementary Materials

Figure 1S. ^{13}C NMR spectrum of polyethylene obtained by catalyst **2**.

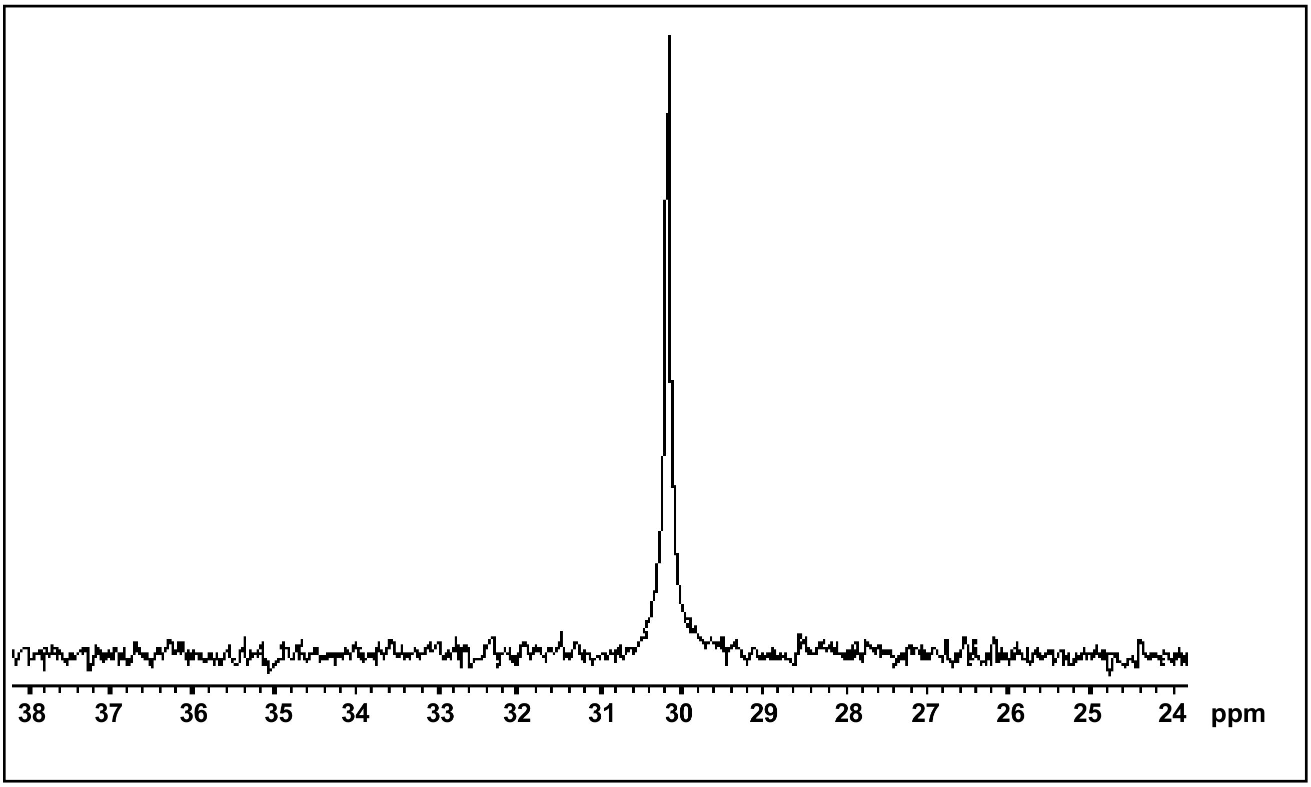

Figure 2S. (**a**–**c**). Mass spectra of *{2,6-[(3,5-ph$_2$pz)CH$_2$]$_2$py}VCl$_3$* (**1**).

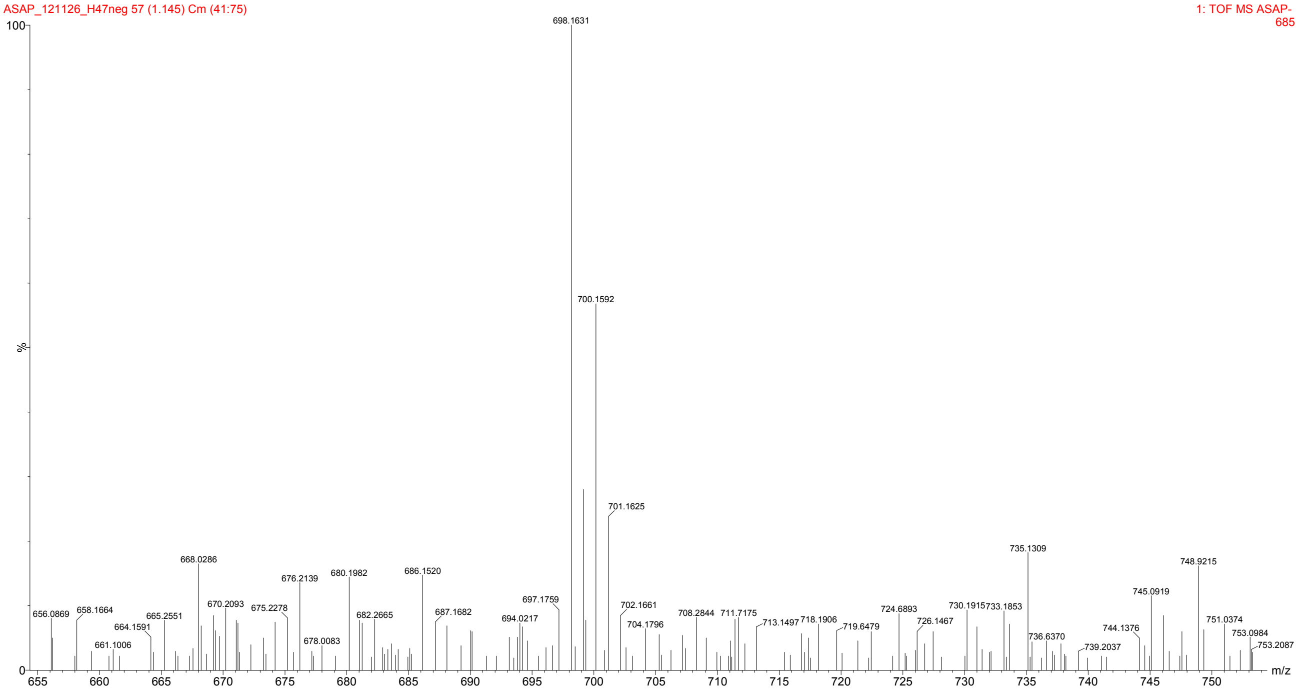

(a)

Figure 2S. *Cont.*

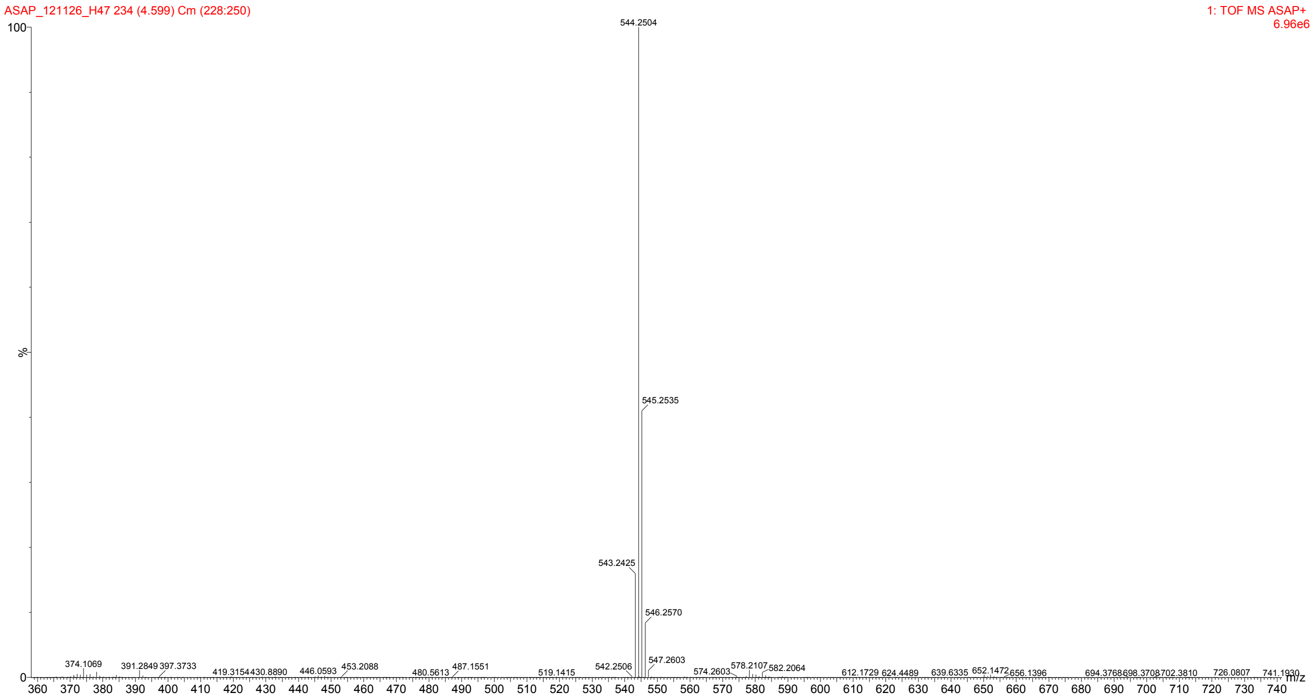

(b)

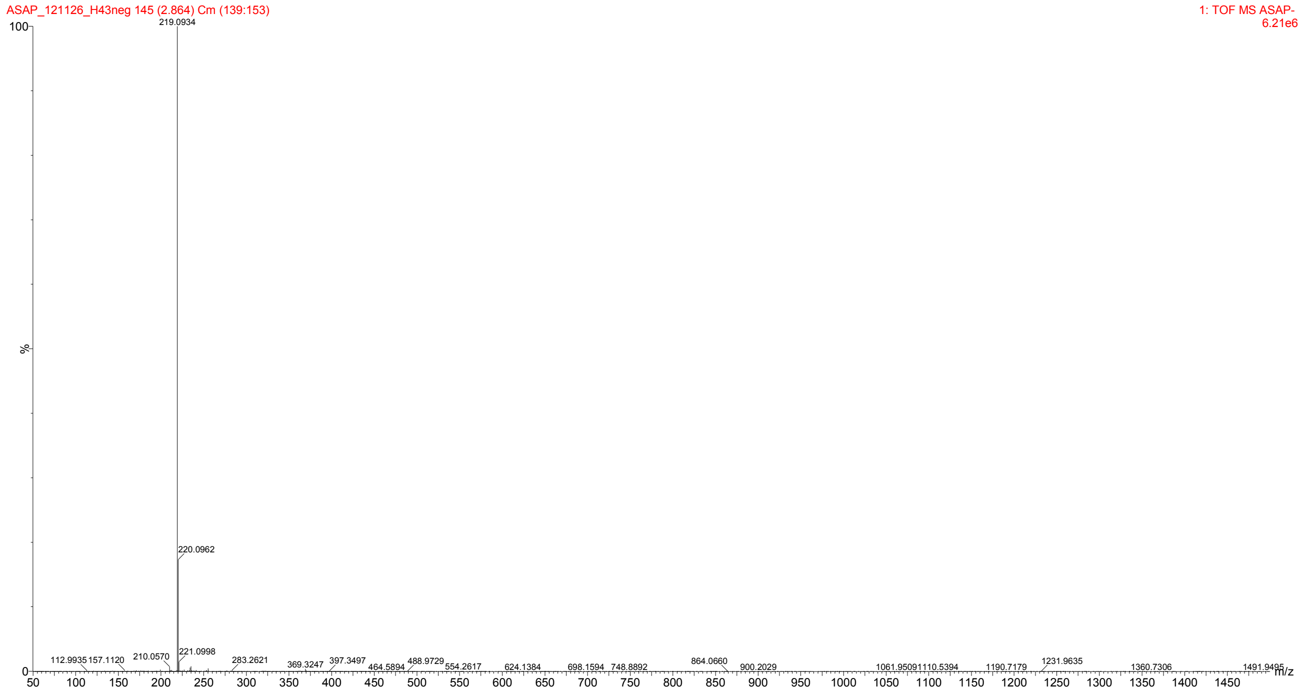

(c)
